# Mood and Changes in Alcohol Consumption in Young Adults during COVID-19 Lockdown: A Model Explaining Associations with Perceived Immune Fitness and Experiencing COVID-19 Symptoms

**DOI:** 10.3390/ijerph181910028

**Published:** 2021-09-24

**Authors:** Agnese Merlo, Noortje R Severeijns, Sarah Benson, Andrew Scholey, Johan Garssen, Gillian Bruce, Joris C Verster

**Affiliations:** 1 Division of Pharmacology, Utrecht Institute for Pharmaceutical Sciences, Utrecht University, 3584CG Utrecht, The Netherlands; a.merlo@uu.nl (A.M.); n.r.severeijns@students.uu.nl (N.R.S.); j.garssen@uu.nl (J.G.); 2 Centre for Human Psychopharmacology, Swinburne University, Melbourne, VIC 3122, Australia; sarahbenson@swin.edu.au (S.B.); andrew@scholeylab.com (A.S.); 3 Division of Psychology and Social Work, School of Education and Social Sciences, University of the West of Scotland, Paisley PA1 2BE, UK; gillian.bruce@uws.ac.uk

**Keywords:** COVID-19, alcohol consumption, immune fitness, stress, mood, social isolation

## Abstract

The aim of this study was to explore the effects of the 2019 Coronavirus (COVID-19) lockdown on mood states, stress, alcohol consumption and perceived immune fitness in a Dutch sample. Analysis included a subsample from the “Corona Lockdown: how fit are you?” (CLOFIT) study, comprising *N* = 761 participants who reported consuming alcohol in 2020. Results show that, compared to pre-lockdown, the first COVID-19 lockdown (March 2020) was associated with experiencing poorer mood (e.g., anxiety, depression, loneliness, fatigue) and increased stress levels. Among younger participants (18 to 35 years old), a significant decrease in weekly alcohol consumption was found during COVID-19 lockdown, which was not significant in older individuals. For the younger age cohort (18 to 35 years old), increased stress significantly correlated to increased weekly alcohol consumption (r = 0.163, *p* = 0.003), which in turn, correlated significantly to reporting a poorer perceived immune fitness (r = −0.165, *p* = 0.002). Poorer perceived immune fitness correlated significantly with increases in the presence and severity of COVID-19 symptoms (r = −0.313, *p* < 0.001, and r = −0.325, *p* < 0.001, respectively). The data provides evidence for significant relationships between changes in mood, stress and alcohol consumption during COVID-19 lockdown, and supports a model that links these changes to perceived immune fitness and susceptibility to experiencing COVID-19 symptoms.

## 1. Introduction

On 11 March 2020, the World Health Organization (WHO) officially declared the 2019 Coronavirus (COVID-19) a pandemic [1]. Due to the nature of the transmission of this virus, multiple actions have been taken since its outbreak to prevent further spread of COVID-19. Important and effective measures were implemented, such as mandatory or advisory (partial) lockdown periods (i.e., stay at home), as well as quarantine for infected individuals (i.e., those experiencing symptoms and those suspected of infection). These measures were introduced with the aim to limit interpersonal contacts in order to reduce virus transmission. Across countries, during lockdown periods people were usually allowed to leave home for essential shopping only (e.g., grocery and pharmacy visits), had to work from home where possible, and were not allowed to socialize indoors with people from other households. Although these measures were necessary and were implemented to reduce transmission rates and save lives, previous research related to quarantine and self-isolation revealed that these solutions may have a significant negative impact on an individuals’ wellbeing, including greater stress levels, depression and low mood [2].

With regards to COVID-19, several studies have reported poorer mood and increased stress during lockdown. For example, Ingram and colleagues [3] have reported lifestyle changes in relation to negative mood. Specifically, a greater negative mood score was observed among participants who had to self-isolate, and was associated with poorer diet, sleep quality and physical activity during COVID-19 lockdown. Another study among 602 Italian participants described that on average almost two-thirds of participants reported lower mood during lockdown, including depressed mood and anxious feelings [4].

Research has shown that individuals apply a variety of measures to cope with self-isolation, which are not always beneficial, and can have an adverse impact on psychological wellbeing. These include changes in sleeping and eating patterns, as well as increased use of tobacco, alcohol and other drugs [5,6]. The current analysis will specifically investigate alcohol consumption before and during lockdown in The Netherlands, as it has been suggested that pandemic-related isolation and stress can act as trigger for increased alcohol consumption [7]. This is also supported by increased alcohol sales data [8,9], despite the closure of pubs and restaurants. On the other hand, it is important to recognize that there is also evidence for a reduction in the use of alcohol as a result of restrictions around public and work gatherings, as well as closure of hospitality venues and changes in living conditions [10,11]. Given these conflicting consequences of lockdown, it is important to further explore alcohol consumption during lockdown, and factors such as mood and stress that may influence the frequency and quantity of alcohol intake.

The literature on loneliness and social isolation reveals that alcohol consumption may increase under such circumstances, as people engage in drinking behavior as a coping mechanism to manage the psychological distress they experience [12,13]. There are an increasing number of studies that address changes in alcohol consumption during COVID-19 lockdown [14,15]. For example, Lechner et al. [14] observed an increase in alcohol consumption and an increase in number of drinking occasions among students, due to lockdown and the subsequent closing of U.S. university campuses. Higher levels of depression and anxiety were also present and linked to increased alcohol consumption. Drinking as a coping mechanism during the COVID-19 pandemic was investigated by Rodriguez et al. [8]. They reported an association between drinking alcohol and COVID-19 related stress. Stress was linked to greater maximum number of alcoholic drinks consumed, i.e., greater number of drinks consumed on a typical occasion, and to increased frequency of past month’s drinking days. Overall, the COVID-19 pandemic is related to several factors that may account for increased stress and alcohol consumption including, but not limited to, loneliness, time spent at home and time spent in quarantine or isolation, which are important correlates [10,16,17,18].

Literature shows that negative mood is associated with greater alcohol cravings and alcohol-seeking behavior [19]. In turn, pessimistic emotional and psychological states can promote further alcohol consumption as a way to mitigate and cope with negative mood states and stress [20]. According to Koob and Le Moal’s allostatic model, alcohol dependence is linked to the transition of alcohol being used in relation to enjoyable states and emotions (positive reinforcement) to its use to mitigate adverse and withdrawal states when sober (negative reinforcement) [20].

Increased alcohol consumption may have negative effects on immune fitness, i.e., the capacity of the body to respond to health challenges such as viral infections, making individuals more susceptible to viral exposure and/or infections [21,22,23]. Recent studies have investigated and asserted the adverse effects of alcohol on the immune system and greater risk of respiratory diseases, such as pneumonia and acute respiratory distress syndrome (ARDS) [24]. Moreover, studies have shown that COVID-19 can progress to ARDS and that this is associated with respiratory failure and mortality rates of up to 70% of COVID-19 cases [25,26]. Furthermore, behavioral disinhibition as a result of alcohol ingestion could also play a role in the spread of the virus, as individuals engage in risky behaviors and poor compliance with government guidelines driven by reduced risk perception (e.g., not wearing a face mask or not applying social distancing), exposing themselves to the virus [27,28]. Excessive alcohol consumption has been associated with lung infections and pulmonary disease, ARDS, as well as increased need of ventilation and prolonged stays in intensive care units [29,30]. Previous studies investigating other viral outbreaks such as SARS or Ebola have reported enhanced stress levels in relation to self-isolation, and a corresponding higher risk of increased alcohol consumption [24,31,32].

**Figure 1:**
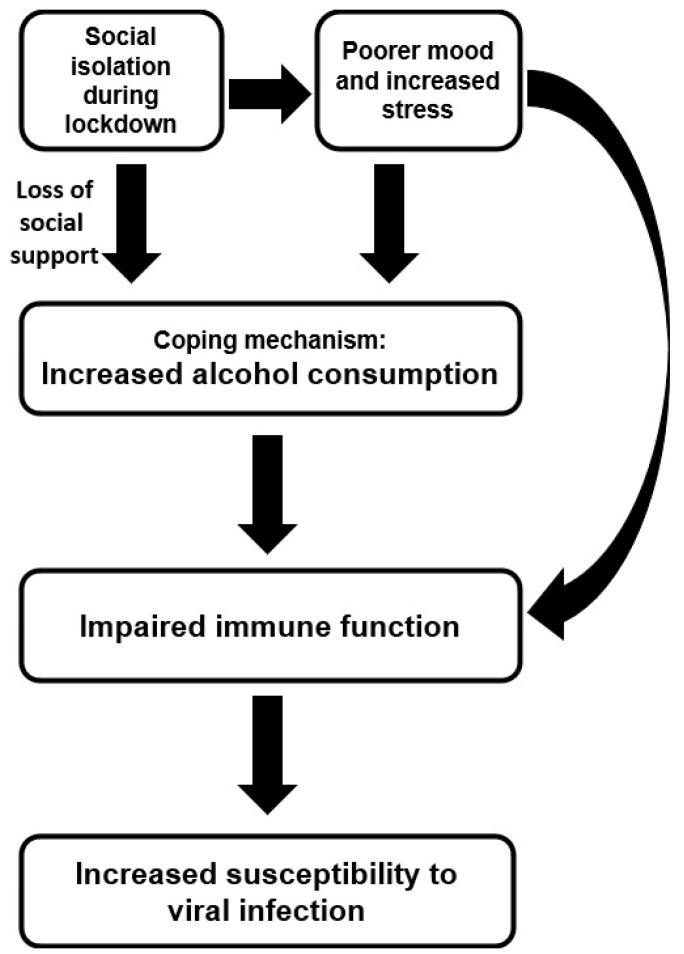
Schematic representation of a model explaining the role of alcohol consumption during COVID-19 lockdown.

Based on the scientific evidence discussed above, in this study a model is proposed that describes the negative impact of lockdown on mood, stress and alcohol consumption, which subsequently has a negative effect on perceived immune fitness and experiencing COVID-19 symptoms (See Figure 1). At a recent webinar by the National Institute on Alcohol Abuse and Alcoholism (NIAAA), a comparable model was presented to explain the potential impact of social isolation on alcohol consumption during the COVID-19 pandemic [21].

In The Netherlands, the first lockdown was enforced from the 15th of March until the 11th of May 2020. The first COVID-19 case in the Netherlands was confirmed on 27 February 2020. The lockdown measures included the closure of schools, and all businesses except supermarkets and pharmacies. People were advised to stay at home as much as possible, in particular when they experienced COVID-19 related health complaints. Except for those with “vital” occupations (e.g., police force, healthcare workers), people were advised to work from home where possible. On 11 May 2020, the lockdown ended and schools and daycare partially reopened, and on 1 June, bars, restaurants, theaters and museums were allowed to reopen, taking into account social distancing and health measures to prevent spreading the virus. At the time the survey was completed, except for large scale events, all businesses were reopened.

The aim of the current analysis was to verify the model summarized in Figure 1, by comparing pre-lockdown and lockdown data on mood, stress, perceived immune fitness and the presence and severity of COVID-19 related symptoms. It was hypothesized that physical distancing and social isolation may result in loneliness, negative mood and increased psychological distress. The potential loss of social support and inadequate coping mechanisms adopted by individuals may lead to increased alcohol consumption.

## 2. Materials and Methods

The current analysis includes data collected from the “Corona Lockdown: how fit are you?” (CLOFIT) study [33]. An online survey was conducted between 24th of June and the 26th of July 2020 to investigate the psychosocial and health consequences of the COVID-19 pandemic in The Netherlands. The survey included questions for the period before the national lockdown (15th of January–14th of March 2020) as well as for the lockdown period (15th of March–11th of May 2020).

The survey was developed in SurveyMonkey and participants’ recruitment was conducted via Facebook advertisement. The study aimed to include the general Dutch population, and its advertisement targeted Dutch adults aged 18 years and older. No other inclusion or exclusion criteria were present. As a form of compensation, participants could enter a prize draw after completion of the survey (one of five EUR 100 vouchers). Electronic informed consent was obtained from all participants and the Ethics Committee of the Faculty of Social and Behavioral Sciences of Utrecht University granted ethical approval for this study (approval code FETC17-061).

### 2.1. Survey Content

Demographics included age, sex, weight and height. Body Mass Index (BMI) was computed by dividing the participants weight (in kilograms) by the square of the person’s height (in meters). Participants reported the number of alcoholic drinks they consumed on average per week and the number of days per week they consumed alcohol. Guidance was provided regarding drink sizes and how to convert these into standardized units of alcohol (10 g ethanol per standard drink). Mood was assessed via 1-item scales and included “anxiety”, “stress”, “depression”, “fatigue”, “hostile”, “loneliness” and “happy” [34]. All items were scored on a scale ranging from 0 (absent) to 10 (extreme). In a similar way, “being active” was assessed. Coping with stress and optimism were assessed with the corresponding subscale of the Fantastic Lifestyle Checklist [35,36]. Scores range from 0 to 8 for coping with stress and 0–4 for optimism, with higher scores implying better coping with stress or being more optimistic.

Perceived immune fitness during lockdown was assessed using a 1-item scale ranging from 0 (poor) to 10 (excellent), with higher scores indicating a better perceived immune fitness [37,38]. The COVID-19 Symptoms Scale comprises nine items, including sneezing, running nose, sore throat, cough, malaise/feeling sick, high temperature (up to 38 Celsius), fever (38 Celsius and higher), shortness of breath and chest pain. The severity of each of the 9 items could be rated as none (0), mild (1), moderate (2) or severe (3). The sum of the item scores is the COVID-19 Symptom severity score, with a possible range from 0 (no complaints) to 27 (severe complaints). In addition, the presence of COVID-19 symptoms was calculated by counting the number of symptoms with a score > 0. Cronbach’s alpha of the COVID-19 Symptoms Scale was 0.817.

### 2.2. Statistical Analysis

Statistical analyses were conducted with SPSS (IBM Corp. Released 2013. IBM SPSS Statistics for Windows, Version 27.0. Armonk, NY: IBM Corp.). Mean (M) and standard deviation (SD) were computed for all variables. Normality was checked statistically (Kolmogorov–Smirnov test) and by visual inspection. Alcohol consumption data before and during lockdown were not normally distributed and therefore were compared using the Related Samples Wilcoxon Signed Rank Test. Differences are considered statistically significant if *p* < 0.01 (two-tailed). To evaluate the impact on mood changes during lockdown, difference, i.e., delta, scores (during lockdown–before lockdown) were computed for alcohol consumption outcomes and mood assessments. The difference scores were normally distributed and correlated using Pearson’s correlations. Correlations are considered statistically significant if *p* < 0.01.

## 3. Results

*N* = 1910 participants started the survey, and after reading informed consent 95.9% of them agreed to participate. Of these *N* = 761 participants consumed alcohol before and during lockdown (39.8%). This sample had an age range of 18 to 94 years old, and 61.6% of them were women. Participant demographics are summarized in Table 1.

Table 2 summarizes alcohol consumption outcomes of the sample. No significant differences were found in alcohol consumption before and during lockdown.

However, further analysis revealed that outcomes differed according to age and sex.

When analyzing the data according to sex, it appeared that men consumed significantly more alcohol per week than women both before lockdown (mean (SD): 9.2 (11.4) versus 3.9 (4.8), *p* < 0.001) and during lockdown (mean (SD): 9.0 (12.7) versus 3.8 (4.9), *p* < 0.001). Men also reported significantly more drinking days per week before lockdown (mean (SD): 3.1 (2.3) versus 2.0 (1.8), *p* < 0.001) and during lockdown (mean (SD): 3.0 (2.4) versus 2.1 (2.0), *p* < 0.001) than women.

When analyzing the data according to age bins (see Table 3) it appeared that above the age of 35, weekly alcohol consumption was unchanged. However, in the younger age bins, during lockdown a significant reduction in weekly alcohol consumption was found for 18- to 25-year-old participants was found, whereas the number of drinking days significantly increased for the 26- to 35-year-old age-group. Across all age groups, significant sex differences were found in that women consume significantly less alcohol than men (data not shown), but the interactions between sex and age-group were not statistically significant.

For the age group 36 and older no effects on alcohol consumption (frequency and quantity) were found, whereas the 18- to 35-year-old drinkers showed a significant overall decrease in weekly alcohol consumption (mean (SD) number of weekly alcoholic drinks before versus during lockdown were 5.2 (6.5) and 4.6 (6.5), respectively, *p* = 0.030), and a significant overall increase in weekly drinking days (mean (SD) number of drinking days before versus during lockdown were 1.8 (1.3) and 1.9 (1.7), respectively, *p* = 0.044). Given the fact that changes in alcohol consumption were only seen in the younger age groups, the following analyses and validation of the model explaining the relation of alcohol consumption on perceived immune fitness and experiencing COVID-19 symptoms was conducted using data only from the younger age groups (18- to 35-year-old participants).

The study outcomes for 18- to 35-year-old participants, summarized in Table 4, show that during lockdown mood was significantly poorer compared to before lockdown. This was accompanied by significantly higher stress levels and poorer perceived immune fitness, but not by a significant increase in reported COVID-19 symptoms.

Although an overall decrease in weekly alcohol consumption was found, it must be noted that about one third of 18- to 35-year-old participants did not change their alcohol consumption during lockdown (30.7%), 38.3% reduced their weekly alcohol intake and 31.0% increased weekly alcohol consumption. Therefore, difference scores (Δ, during lockdown–before lockdown) were computed to relate changes in weekly alcohol consumption to other study outcomes. Pearson’s correlations between changes in mood and the other study outcomes are listed in Table 5.

All mood assessments correlated significantly with each other (*p* < 0.001). All mood assessments also correlated significantly with perceived immune fitness, but correlations between mood and the presence or severity of COVID-19 were not statistically significant. No relevant sex differences were found.

The relationship of stress and coping with stress with other study outcomes is summarized in Table 6. Higher levels of stress during lockdown were associated with significantly increased weekly alcohol intake (r = 0.163, *p* = 0.003). The correlation between Δ stress and number of drinking days did not reach significance (r = 0.106, *p* = 0.052). A higher level of coping with stress was associated with significantly reduced stress (r= −0.544, *p* < 0.001), significantly reduced weekly alcohol intake (r = −0.225, *p* = 0.002) and a significantly reduced number of drinking days during lockdown (r = −0.279, *p* < 0.001). Coping with stress was positively correlated with perceived immune fitness (*p* < 0.001), and the association approached significance for the presence and severity of COVID-19 symptoms (*p* = 0.012 and *p* = 0.028, respectively). For men only, the correlations between coping with stress and the presence (*p* = 0.005) and severity (*p* = 0.006) of COVID-19 symptoms were statistically significant.

**Figure 2:**
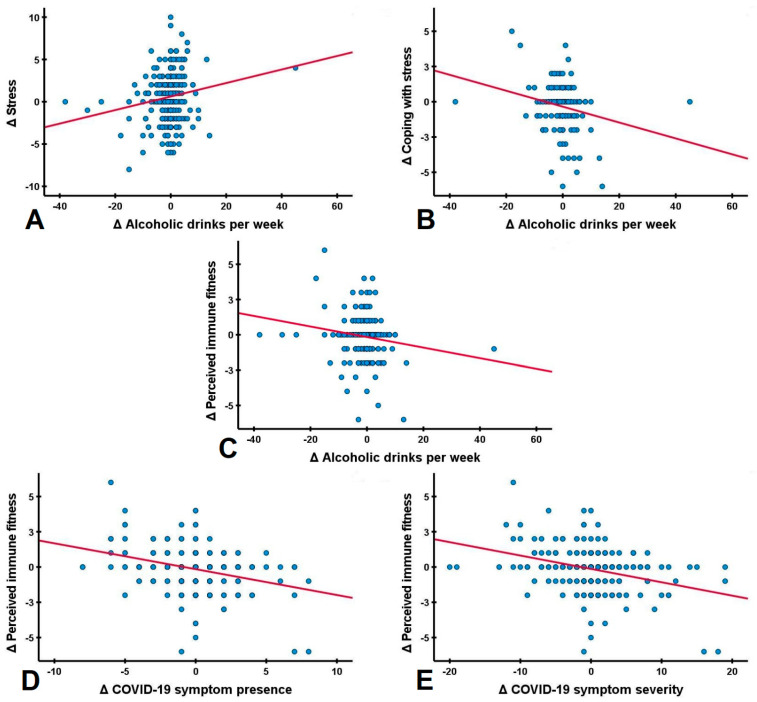
Correlations between changes in stress, alcohol consumption, perceived immune fitness and the presence and severity of COVID-19 symptoms during the COVID-19 lockdown. Pearson correlations (red lines) of difference scores (Δ, during COVID-19 lockdown versus before COVID-19 lockdown) are shown for (**A**) stress and alcoholic drinks per week; (**B**) coping with stress and alcoholic drinks per week; (**C**) alcoholic drinks per week and perceived immune fitness; (**D**) perceived immune fitness and the presence of COVID-19 symptoms and (**E**) perceived immune fitness and the severity of COVID-19 symptoms. All correlations were statistically significant (*p* < 0.01).

The relationship between perceived immune fitness and COVID-19 symptoms with alcohol consumption is summarized in Table 7. Weekly alcohol consumption was significantly correlated with experiencing a poorer immune fitness during lockdown (r = −0.165, *p* = 0.002). However, the correlation between Δ weekly drinking days and Δ perceived immune fitness was not significant (r= −0.96, *p* = 0.113). Additionally, direct correlations between alcohol consumption outcomes and the presence and severity of COVID-19 were not significant. Separate analyses for men and women did not reveal significant correlations. However, significant correlations were found between perceived immune fitness and COVID-19 symptom severity (r = −0.325, *p* < 0.001) and between perceived immune fitness and the number of reported COVID-19 symptoms (r = −0.313, p < 0.001). Taken together, these correlational analyses support the model proposed in the Introduction Section of this article (See Figure 2).

## 4. Discussion

The objective of this study was to evaluate the proposed model describing the relationship between alcohol consumption and susceptibility to COVID-19 symptoms (see Figure 1). Our data of young adults demonstrate the adverse role of lockdown in aggravating mood and increasing stress levels. Increased alcohol consumption correlated significantly with reporting poorer perceived immune fitness, as well as a higher frequency and severity of experienced COVID-19 symptoms.

Our findings are in line with recent publications on alcohol consumption during lockdown. However, scientific literature shows mixed results. Several studies investigating alcohol consumption during the pandemic suggest that psychological distress related to COVID-19 is linked to changes in drinking behaviors, including heavy drinking (more units per occasion) and increased frequency of consumption [8,39,40,41,42]. However, other studies reported no change in alcohol consumption or, similarly to the current study, a reduction in alcohol consumption [41,42,43,44]. The discrepancy in study outcomes suggests that the observed alcohol effects of the various studies depend on the demographic characteristics of the convenience sample that was included [40,43,44]. For example, the reduction seen in alcohol consumption among younger participants was not seen among those aged 36 years and older.

It was found that increased alcohol consumption, but not the number of drinking days, was associated with poorer perceived immune fitness. Finally, perceived immune fitness correlated significantly with the presence and severity of COVID-19 symptoms. The latter is not surprising, as COVID-19 is a disease that affects immune functioning. Taken together, these findings support the proposed model summarized in Figure 1.

It should be noted however that although the observed correlations were significant, they were usually of moderate magnitude. This modest effect size is most likely due to the fact that the sample is not homogenous: about one third of the participants (30.7%) did not alter their weekly alcohol consumption during lock-down, whereas 38.3% of drinkers reported a reduction and 31.0% reported an increase in weekly alcohol consumption. A detailed comparison of these groups is presented elsewhere [45]. Including the relative large number of participants in the analysis that did not alter their drinking behavior has a lowering effect on the observed correlations.

The findings should be viewed in the context of the study’s limitations. Firstly, the data collected are self-reported, and thus responses could potentially be inaccurate and impacted by recall bias. In this context, it should be noted that the time interval between data collection and the assessed (pre)-lockdown periods was three to six months. Nevertheless, lockdown is an exceptional period, and as such individuals should be able to recall the impact relatively well. On the other hand, individuals may have exaggerated the lockdown effects or idealized the period prior to the lockdown. Other prospective studies are underway to verify these assumptions. Secondly, we assessed perceived immune fitness, a self-assessment of the body’s capacity to respond to health challenges [37]. Although this observational measure is adequate for clinical settings and research purposes [37], in the current study this subjective measure was not supported by additional objective assessments of immune functioning, such as changes in cytokines or other immune biomarkers. Future studies should assess these biomarkers to strengthen the support for the proposed model in Figure 1.

Thirdly, while the presence and severity of COVID-19 symptoms were assessed, at the time the survey was conducted most Dutch individuals were not actually tested for infection with SARS-CoV-2. Additionally, in the current sample, 77.2% of the participants had not been tested for COVID-19, and of those that were tested the vast majority (38 of 46 participants) tested negative (i.e., were not infected). Therefore, while poorer immune fitness correlated significantly with increased presence and severity of COVID-19 symptoms, the current data do not allow any causal inferences. Future studies should relate findings to COVID-19 screening test results. The latter is also important in the context that individuals may test positive for COVID-19 while being asymptomatic, i.e., not reporting any COVID-19 symptoms.

Fourthly, the COVID-19 Symptoms Scale used in this study was designed in the first quarter of 2020. It includes symptoms that at that time were outlined by the Dutch National Institute for Public Health and the Environment (RIVM) [46]. However, to date (the first quarter of 2021) it is known that this symptom listing was incomplete, and thus several symptoms that are currently recognized as relevant symptoms were not evaluated (e.g., loss of taste and smell). Notwithstanding this, significant correlations were found between poorer perceived immune fitness and increased reporting of the presence and severity of COVID-19 symptoms. Future studies should, however, update the COVID-19 symptom listing.

Fifthly, the model proposed in this article describes the impact of changes in alcohol consumption on perceived immune fitness and reporting COVID-19 related symptoms. There are however many other variables that also may have an impact on perceived immune fitness and the presence and severity of COVID-19 symptoms. These include, for example, underlying disease, and perhaps also demographics such as socioeconomic status and educational level. It was beyond the scope of this article to present these analyses here. However, future analysis should determine the impact of these variables. Sixthly, the data were collected via Facebook. This may have influenced recruitment as not everybody uses this social media platform. Therefore, although the obtained sample size is large and diverse in terms of age and sex, and provides confidence in the presented observations, it is not established that this convenience sample is also a nationally representative sample. Lastly, it is important to note that the statistics to support the model comprise correlational analysis. Therefore, causality cannot be inferred by these findings, and directional conclusions cannot be drawn. Prospective studies are needed to further validate the model and verify our findings.

Our findings are of importance for community and policy practice as they demonstrate the link between increased alcohol consumption and experiencing poorer immune fitness and the increase in experiencing COVID-19 related symptoms. Lockdown can have a significant negative effect on mood and health, which is sometimes overseen by policy makers in their efforts to counteract the spread of the virus. Individuals may suffer from lockdown effects such as loneliness, stress and depressive feelings. Furthermore, as a strategy to cope with these they may engage in different mechanisms, and increased alcohol consumption may be one of these. Closure of pubs and restaurants and stay at home orders further limit opportunities to monitor individuals’ drinking behavior. The community should be informed about the negative consequences of increased alcohol consumption in relation to maintaining adequate immune fitness and the susceptibility to experiencing COVID-19 related symptoms. Improving immune fitness and strengthening coping strategies to manage stress and mood changes may help reduce the susceptibility to developing COVID-19 symptoms and reduce their severity.

## 5. Conclusions

Although national lockdowns, self-isolation and social distancing are essential measures to reduce and prevent the spread of the COVID-19, their impact on individuals’ wellbeing must be carefully considered and appropriately addressed to inform strategies for minimizing adverse psychological and mood outcomes. The current article presents data that support a model showing how poorer mood and increased stress during lockdown are associated with increased alcohol consumption, poorer perceived immune functioning and the increased presence and severity of COVID-19 symptoms.

## Figures and Tables

**Table 1:** Demographics and study outcomes.

	Overall	Men	Women	*p*-Value
*N* (%)	761 (100%)	292 (38.4%)	469 (61.6%)	
Age (year)	42.3 (19.0)	48.0 (19.2)	38.7 (18.0) *	<0.001 *

Mean and standard deviation (SD, between brackets) are shown. Significant differences between men and women (*p* < 0.01) are indicated by *. Abbreviations: BMI = body mass index.

**Table 2:** Alcohol consumption before and during lockdown.

Variables Assessed	Before Lockdown	During Lockdown	*p*-Value
Alcoholic drinks/week	6.0 (8.5)	5.9 (9.2)	0.310
Drinking days/week	2.4 (2.1)	2.5 (2.2)	0.179

Mean and standard deviation (SD, between brackets) are shown.

**Table 3:** Alcohol consumption before and during lockdown according to age.

Variable	*N*	Before Lockdown			During Lockdown			*p*-Value (Before vs. During Lockdown)		
**Weekly alcohol consumption**		Men	Women	Overall	Men	Women	Overall	Men	Women	Overall
18–25 years old	219	10.2 (9.3)	3.9 (5.0) ^†^	5.5 (7.0)	7.6 (10.7)	3.4 (4.2) ^†^	4.5 (6.8)	0.001 *	0.395	0.003 *
26–35 years old	120	6.3 (7.2)	3.8 (4.1)	4.6 (5.5)	6.6 (8.6)	3.7 (3.8)	4.7 (6.1)	0.597	0.716	0.568
36–45 years old	54	14.6 (17.5)	3.3 (3.2) ^†^	8.1 (12.8)	13.6 (15.3)	4.2 (4.8)	8.2 (11.5)	0.833	0.060	0.116
46–55 years old	88	5.6 (6.8)	3.9 (5.6)	4.6 (6.2)	7.3 (9.5)	3.8 (5.1)	5.4 (7.6)	0.243	1.000	0.446
56–65 years old	128	9.9 (12.4)	4.2 (5.4) ^†^	6.7 (9.5)	11.0 (16.3)	4.0 (6.4) ^†^	7.0 (12.2)	0.314	0.383	0.985
6–75 years old	85	10.1 (13.9)	5.3 (5.2)	8.4 (11.9)	9.8 (14.5)	5.8 (6.0)	8.4 (12.3)	0.582	0.306	0.888
≥75 years old	19	7.4 (6.8)	2.9 (2.7)	5.3 (5.6)	7.5 (6.8)	3.2 (3.2)	5.5 (5.7)	0.317	0.180	0.102
**Drinking days per week**										
18–25 years old	219	2.4 (1.5)	1.5 (1.2) ^†^	1.7 (1.3)	2.2 (1.8)	1.6 (1.5)	1.8 (1.6)	0.274	0.159	0.627
26–35 years old	120	2.1 (1.7)	1.6 (1.1)	1.8 (1.3)	2.4 (2.0)	2.0 (1.7)	2.2 (1.8)	0.074	0.030	0.005 *
36–45 years old	54	3.4 (2.5)	2.1 (1.8)	2.6 (2.2)	3.3 (2.4)	2.5 (2.2)	2.9 (2.3)	0.931	0.114	0.192
46–55 years old	88	2.0 (2.2)	2.1 (2.0)	2.1 (2.1)	2.3 (2.5)	2.0 (2.1)	2.1 (2.3)	0.336	0.360	0.934
56–65 years old	128	3.7 (2.3)	2.6 (2.3) ^†^	3.1 (2.3)	3.8 (2.5)	2.4 (2.4) ^†^	3.0 (2.5)	0.670	0.307	0.566
66–75 years old	85	4.2 (2.5)	3.5 (2.5)	4.0 (2.5)	3.7 (2.6)	3.8 (2.6)	3.7 (2.6)	0.011	0.236	0.131
≥ 75 years old	19	4.1 (2.7)	2.8 (2.8)	3.5 (2.8)	4.1 (2.8)	2.8 (2.8)	3.5 (2.8)	1.000	1.000	1.000

Mean and standard deviation (SD, between brackets) are shown. Significant differences (*p* < 0.01) between before and during lockdown are indicated by *. Significant sex differences (*p* < 0.01) are indicated by †.

**Table 4:** Means and standard deviations for assessments before and during lockdown.

Variables Assessed	Before Lockdown			During Lockdown			*p*-Value (Before vs. During Lockdown)		
	Men	Women	Overall	Men	Women	Overall	Men	Women	Overall
Anxiety	1.6 (1.9)	2.1 (2.5)	2.0 (2.3)	2.7 (2.6)	3.2 (2.8)	3.1 (2.7)	<0.001 *	<0.001 *	<0.001 *
Depression	1.5 (1.9)	2.2 (2.4) ^†^	2.0 (2.3)	2.5 (2.6)	3.3 (3.0)	3.1 (2.9)	<0.001 *	<0.001 *	<0.001 *
Loneliness	1.8 (2.1)	2.1 (2.2)	2.0 (2.2)	3.6 (2.9)	3.7 (2.9)	3.7 (2.9)	<0.001 *	<0.001 *	<0.001 *
Fatigue	3.7 (2.6)	4.9 (2.6) ^†^	4.5 (2.7)	3.9 (2.6)	4.9 (2.5) ^†^	4.6 (2.6)	0.406	0.652	0.418
Hostile	0.9 (1.5)	0.9 (1.8)	0.9 (1.7)	1.6 (2.4)	1.2 (2.2)	1.3 (2.2)	<0.001 *	<0.001 *	<0.001 *
Happy	7.3 (1.4)	6.9 (1.7)	7.0 (1.6)	6.4 (1.7)	6.0 (2.0)	6.1 (1.9)	<0.001 *	<0.001 *	<0.001 *
Stress	3.5 (2.6)	4.6 (2.5) ^†^	4.3 (2.6)	4.2 (2.7)	5.1 (2.5) ^†^	4.8 (2.6)	0.010	0.005 *	<0.001 *
Optimism	3.1 (0.8)	2.9 (1.0)	3.0 (1.0)	2.8 (0.8)	2.6 (1.0)	2.6 (1.0)	0.025	<0.001 *	<0.001 *
Being active	6.9 (2.0)	6.7 (2.0)	6.8 (2.0)	5.5 (2.2)	5.5 (2.3)	5.5 (2.3)	<0.001 *	<0.001 *	<0.001 *
Coping with stress	6.0 (1.6)	5.3 (1.7)	5.5 (1.7)	5.6 (1.8)	5.0 (1.8)	5.1 (1.8)	0.026	0.029	0.003 *
Perceived immune fitness	7.8 (1.5)	7.4 (1.5)	7.5 (1.5)	7.5 (1.6)	7.3 (1.6)	7.4 (1.6)	0.012	0.286	0.022
COVID-19 symptoms severity	3.0 (3.1)	4.3 (4.0) ^†^	3.9 (3.8)	3.0 (3.4)	4.4 (4.2) ^†^	4.0 (4.1)	0.538	0.474	0.737
COVID-19 symptoms presence	2.3 (2.0)	3.0 (2.2)	2.8 (2.2)	2.3 (2.0)	2.9 (2.2) ^†^	2.7 (2.1)	0.438	0.915	0.645

Mean and standard deviation (SD, between brackets) are shown. Significant differences (*p* < 0.01) between before and during lockdown are indicated by *. Significant sex differences (*p* < 0.01) are indicated by †.

**Table 5:** Relationship of mood with other study outcomes.

Variables	Sex	Δ Stress	Δ Coping with Stress	Δ Alcoholic Drinks/Week	Δ Drinking Days/Week	Δ Perceived Immune Fitness	Δ COVID-19 Symptoms Severity	Δ COVID-19 Symptoms Presence
Δ Anxiety	Men	0.503 *	−0.309	0.064	0.242	−0.343 *	−0.024	−0.088
	Women	0.535 *	−0.433 *	0.128	0.007	−0.256 *	0.044	0.016
	Overall	0.528 *	−0.407 *	0.088	0.075	−0.278 *	0.029	−0.010
Δ Depression	Men	0.339 *	−0.604 *	0.058	0.059	−0.436 *	0.199	0.167
	Women	0.522 *	−0.528 *	0.157	0.167	−0.368 *	0.071	0.099
	Overall	0.482 *	−0.535 *	0.106	0.138	−0.375 *	0.092	0.111
Δ Loneliness	Men	0.185	−0.109	−0.141	0.054	−0.242	0.086	0.076
	Women	0.277 *	−0.377 *	−0.021	0.035	−0.176 *	0.076	0.062
	Overall	0.252 *	−0.318 *	−0.075	0.038	−0.195 *	0.077	0.066
Δ Fatigue	Men	0.429 *	−0.421 *	0.161	0.112	−0.152	0.063	0.039
	Women	0.492 *	−0.561 *	0.146	0.133	−0.382 *	0.141	0.158
	Overall	0.477 *	−0.530 *	0.135	0.122	−0.327 *	0.123	0.128
Δ Hostile	Men	0.171	−0.320	−0.136	0.003	−0.143	−0.011	−0.013
	Women	0.338 *	−0.327 *	0.069	0.106	−0.166	0.049	0.067
	Overall	0.284 *	−0.326 *	−0.047	0.058	−0.162 *	0.029	0.040
Δ Happy	Men	−0.400 *	0.210	−0.076	0.064	−0.451 *	−0.144	−0.086
	Women	−0.534 *	0.567 *	−0.071	−0.101	0.311 *	−0.080	−0.091
	Overall	−0.503 *	0.496 *	−0.064	−0.089	0.339 *	−0.092	−0.090
Δ Optimistic	Men	−0.530 *	0.467 *	−0.155	−0.258	0.396 *	−0.109	−0.152
	Women	−0.522 *	0.569 *	−0.195	−0.164	0.257 *	−0.065	−0.090
	Overall	−0.524 *	0.544 *	−0.157	−0.190	0.280 *	−0.075	−0.104
Δ Being active	Men	−0.105	0.288	−0.064	−0.182	0.280 *	0.004	0.069
	Women	−0.272 *	0.421 *	−0.042	−0.065	0.435 *	−0.123	−0.075
	Overall	−0.227 *	0.385 *	−0.046	−0.100	0.393 *	−0.090	−0.035

Pearson’s correlations were computed between difference scores (Δ, during lockdown–before lockdown). Significant correlations (*p* < 0.006, two-tailed, after Bonferroni’s correction) are indicated by *.

**Table 6:** Relationship of stress and coping with stress with other study outcomes.

	Δ Stress			Δ Coping with Stress		
	Men	Women	Overall	Men	Women	Overall
Δ Alcoholic drinks/week	0.192	0.169 *	0.163 *	−0.156	−0.351 *	−0.225 *
Δ Drinking days/week	0.105	0.110	0.106	−0.265	−0.298 *	−0.279 *
Δ Perceived immune fitness	−0.321 *	−0.271 *	−0.285 *	0.470 *	0.394 *	0.411 *
Δ COVID-19 symptoms severity	−0.044	0.094	0.061	−0.379 *	−0.096	−0.160
Δ COVID-19 symptoms presence	−0.078	0.127	0.074	−0.387 *	−0.120	−0.183

Pearson’s correlations were computed between difference scores (Δ, during lockdown–before lockdown). Correlations are considered significant if *p* < 0.01 (two-tailed).

**Table 7:** Relationship between changes in perceive immune fitness and COVID-19 symptoms with alcohol consumption outcomes.

	Δ Alcoholic Drinks/Week			Δ Drinking Days/Week		
	Men	Women	Overall	Men	Women	Overall
Δ Perceived immune fitness	−0.221	−0.149	−0.165 *	0.009	−0.137	−0.086
Δ COVID-19 symptoms severity	0.053	−0.002	0.020	−0.186	0.014	0.037
Δ COVID-19 symptoms presence	0.029	0.049	0.037	−0.161	0.024	−0.033

Pearson’s correlations were computed between difference scores (Δ, during lockdown–before lockdown). Correlations are considered significant if *p* < 0.01 (two-tailed).

## Data Availability

The data are available upon request from the corresponding author.
